# PRMT5 competitively binds to CDK4 to promote G1-S transition upon glucose induction in hepatocellular carcinoma

**DOI:** 10.18632/oncotarget.12351

**Published:** 2016-09-30

**Authors:** Hao Yang, Xiaoping Zhao, Li Zhao, Liu Liu, Jiajin Li, Wenzhi Jia, Jianjun Liu, Gang Huang

**Affiliations:** ^1^ Institute of Health Sciences, Shanghai Institutes for Biological Sciences (SIBS), Chinese Academy of Sciences (CAS) & Shanghai Jiao Tong University School of Medicine (SJTUSM), Shanghai 200031, China; ^2^ Department of Nuclear Medicine, Renji Hospital, Shanghai Jiao Tong University School of Medicine, Shanghai 200127, China; ^3^ Institute of Clinical Nuclear Medicine, Renji Hospital, Shanghai Jiao Tong University School of Medicine, Shanghai 200127, China; ^4^ Shanghai University of Medicine and Health Sciences, Shanghai 201318, China

**Keywords:** glucose, PRMT5, cell cycle progression, competitive binding, hepatocellular carcinoma

## Abstract

Although cancer cells are known to be “addicted” to glucose, the effect of glucose in proliferation of these cells remains elusive. Here, we report that upon glucose induction, protein arginine methyltransferase 5 (PRMT5) exerts a profound effect on the G1-S cell cycle progression via directly interacting with cyclin dependent kinase 4 (CDK4) in hepatocellular carcinoma (HCC). Upregulation of both PRMT5 and CDK4 predicts more malignant characteristics in human HCC tissues. Mechanistically, glucose promotes the interaction between PRMT5 and CDK4, which leads to activation of CDK4-RB-E2F-mediated transcription via releasing CDKN2A from CDK4. Moreover, the PRMT5 competitive inhibition of the interaction between CDK4 and CDKN2A is important for glucose-induced growth of HCC cells. Furthermore, the CDK4 mutant R24A weakly binds to PRMT5, inhibiting HCC cell cycle progression and tumor growth. Thus, our findings uncover a critical function for PRMT5 and CDK4 and provide an improved therapeutic strategy against HCC.

## INTRODUCTION

Liver cancer, especially hepatocellular carcinoma (HCC), causes high morbidity and mortality relative to other cancers [1]. Major precipitating factors for hepatocarcinogenesis include hepatitis B virus (HBV) or hepatitis C virus (HCV) infection, chemical cytotoxicity and aberrant hepatic metabolism [2–4]. Although hepatectomy has been identified as the main treatment strategy for HCC, poor prognosis still frequently exists [5]. Therefore, the need to explore underlying therapeutic options and molecular targets is particularly urgent.

Since aberrant progression of the cyclin dependent kinase (CDK)-driven cell cycle is one of the hallmarks of cancers [6], many studies have focused on these cell cycle regulators as potential targets in HCC. Among the CDKs, cyclin dependent kinase 4 (CDK4) plays a specific role in tumorigenesis and development [7]. Combined with cyclin D (CCND), CDK4 controls cancer cells from the G1 phase to S phase for DNA synthesis and expedites cell proliferation [8]. Of these CCNDs (cyclin D1, D2 and D3), CCND1 is the most pivotal for the interaction with CDK4 in hepatic cell cycle progression [9]. On the other hand, some CDK inhibitory proteins (CKIs), such as CDKN2A, CDKN2B, CDKN2C and CDKN2D, inhibit the function of the CDK4 and CCND complex in cell cycle regulation [8, 10]. Overall, the CDK4 and CDKN2A (p16^INK4a^ as the major isoform) complex is more critical for growth suppression in hepatoma cells [11] or other types of tumor cells [12]. With CCND1 stimulation and CDKN2A inhibition, CDK4 phosphorylates the tumor suppressor retinoblastoma protein (RB) at serine780 and serine795 [13], allowing pRB to activate the transcriptional factor E2F [14]. The transcriptional functions of E2Fs have been fully determined. E2F1, E2F2 and E2F3a are perceived as transcriptional activators, while E2F3b, E2F4, E2F5, E2F6 and E2F7 are considered to be repressors [15]. RB interacts with most activators of E2Fs (E2F1, E2F2, E2F3 and E2F4) and suppresses E2F transcriptional activity [16]. The CDK4-RB-E2F pathway expedites the expression of E2F-responsive genes, such as *cyclin E*, *cyclin A*, *CDK1*, *cell division cycle 6* (*CDC6*), *origin recognition complex* subunit 1 (*ORC1*), *minichromosome maintenance complex component 3* (*MCM3*), *myeloblastosis oncogene* (*MYB*) and *E2F1* [8, 17]. By controlling DNA replication and cell cycle, these genes maintain tumor cells in the state of abnormal cell proliferation.

Protein arginine methyltransferase 5 (PRMT5) is an enzyme which can transfer methyl groups to the arginine residues of histones and some nonhistone proteins, and its methyltransferase activity is necessary for tumor cell proliferation [18]. PRMT5 has been considered as a potential target for cancer due to its function in tumor cell cycle regulation. For example, PRMT5 depletion leads to apoptosis and cell cycle arrest via methylation of tumor suppressor p53 [19]. Furthermore, PRMT5 can upregulate levels of cell cycle regulators in lung cancer, such as CDK4/6 and CCND1/D2/E1 [20]. Although one study has shown cyclin D1/CDK4 to phosphorylate MEP50 and then promote PRMT5 methyltransferase activity [21], the concrete interaction between PRMT5 and CDKs in HCC cell cycle regulation still needs to be addressed.

Here, we confirmed that glucose is indispensable for PRMT5 to facilitate HCC cell growth. Under the high glucose condition, PRMT5-depleted cells were more sensitive to a CDK4 inhibitor. Importantly, we identified a direct glucose-induced interaction between PRMT5 and CDK4. Through that interaction, PRMT5 inhibited the interaction between CDK4 and CDKN2A and then activated the CDK4-RB-E2F pathway in HCC cells under glucose induction. Furthermore, we revealed that the CDK4 mutant R24A weakly bound with PRMT5 and inhibited HCC cell cycle progression. As a result, the protein levels of PRMT5 and CDK4 were found to positively correlate in HCC and stimulate HCC cell proliferation.

## RESULTS

### Protein levels of PRMT5 and CDK4 are positively correlated, which predict more malignant characteristics in human HCC tissues

**Figure 1:**
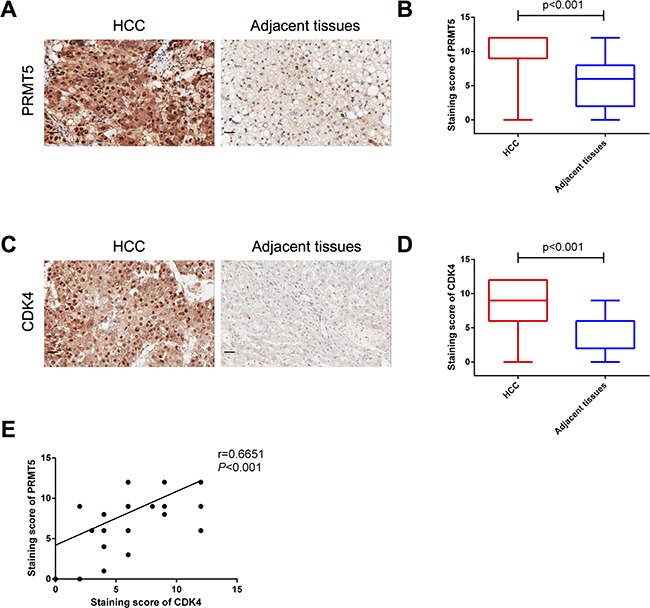
Protein levels of PRMT5 and CDK4 are positively correlated **A**, **C.** Representative histopathologic sections of human HCC and adjacent tissues were stained with PRMT5 (A) and CDK4 (C) antibodies (Scale bar, 20 μm). **B**, **D.** Semi-quantitative immunohistochemical analysis of 75 human HCC and adjacent tissues for PRMT5 (B) and CDK4 (D). The experiments were tested with paired t-test. **E.** Pearson correlative analysis of semi-quantitative staining scores for PRMT5 and CDK4. The standard curve was drawn by linear regression of the correlation scores.

To identify the role of PRMT5 and CDK4 in HCC, we analyzed 75 pairs of human HCC and adjacent tissues by immunohistochemistry (IHC). As shown in Figure 1A, PRMT5 proteins were detected in almost all HCC cells, and quantification of the staining on a scale of 0 to 12 showed that 62 out of 75 (83%) human HCC tissues displayed high PRMT5 expression levels compared with the adjacent normal tissues (Table 1 and Figure 1B). By statistical analysis of clinicopathological parameters of these 75 HCC patients, PRMT5 protein levels were observably correlated with HCC tumor stage (*P* = 0.029). However, patient sex, age, degree of tumor differentiation and other parameters had no observable relationship with PRMT5 expression (Table 1). Analogously, IHC analysis also revealed that CDK4 proteins were markedly detected (Figure 1C) in HCC tissues and highly expressed in 46 (61%) cases (Table 1 and Figure 1D). The tumor size and tumor stage, but not other parameters, correlated with tumor CDK4 expression (*P*<0.05, Table 1 =. Moreover, we divided the 75 HCC samples into four groups: high CDK4 and high PRMT5 expression, low CDK4 and low PRMT5 expression, high CDK4 and low PRMT5 expression, low CDK4 and high PRMT5 expression (the representative samples were shown in Supplementary Figure S1). The statistical analysis of clinicopathological parameters showed a correlation between CDK4 and PRMT5 co-expression level and tumor size or tumor stage (*P* < 0.05, Table 2). Furthermore, we detected a correlation (Pearson r = 0.6651, *P* < 0.001, Figure 1E) between the staining scores of CDK4 and PRMT5 expressed in HCC tissues. Thus, these results indicated that the protein levels of PRMT5 and CDK4 are positively correlated in human HCC tissues, which predict more malignant characteristics.

**Table 1:** Analysis of correlation between CDK4 or PRMT5 protein levels and clinicopathological parameters of HCC patients

Protein Characteristics		CDK4				PRMT5			
		All cases	High	Low	*P*-value	All cases	High	Low	*P*-value
Participants		75	46	29		75	62	13	
Sex					0.816				0.369
	Male	63	39	24		63	51	12	
	Female	12	7	5		12	11	1	
Age					0.497				0.290
	<60 years	55	35	20		55	47	8	
	≥60 years	20	11	9		20	15	5	
Tumor size					0.020				0.216
	≤5cm	29	13	16		29	22	7	
	>5cm	46	33	13		46	40	6	
Tumor stage					0.003				0.029
	I	17	8	9		17	11	6	
	II	26	11	15		26	20	6	
	III	22	20	2		22	21	1	
	IV	10	7	3		10	10	0	
Degree of differentiation					0.219				0.187
	I	3	2	1		3	2	1	
	II	60	34	26		60	48	12	
	III	12	10	2		12	12	0	
Distant metastasis					0.949				0.289
	Positive	5	3	2		5	5	0	
	Negative	70	43	27		70	57	13	

**Table 2:** Analysis of correlation between co-expression levels of CDK4/PRMT5 and clinicopathological parameters of HCC patients

Proteinc Characteristics		All cases	CDK4/PRMT5				*P*-value
			+/+	+/−	−/+	−/−	
Participants		75	45	1	17	12	
Sex							0.696
	Male	63	38	1	13	11	
	Female	12	7	0	4	1	
Age							0.315
	<60 years	55	35	0	12	8	
	≥60 years	20	10	1	5	4	
Tumor size							0.049
	≤5cm	29	12	1	10	6	
	>5cm	46	33	0	7	6	
Tumor stage							0.014
	I	17	7	1	4	5	
	II	26	11	0	9	6	
	III	22	20	0	1	1	
	IV	10	7	0	3	0	
Degree of differentiation							0.504
	I	3	2	0	0	1	
	II	60	33	1	15	11	
	III	12	10	0	2	0	
Distant metastasis							0.651
	Positive	5	3	0	2	0	
	Negative	70	42	1	15	12	

(+) high expression; (−) low expression.

### Glucose-induced PRMT5 promotes HCC cell proliferation

**Figure 2:**
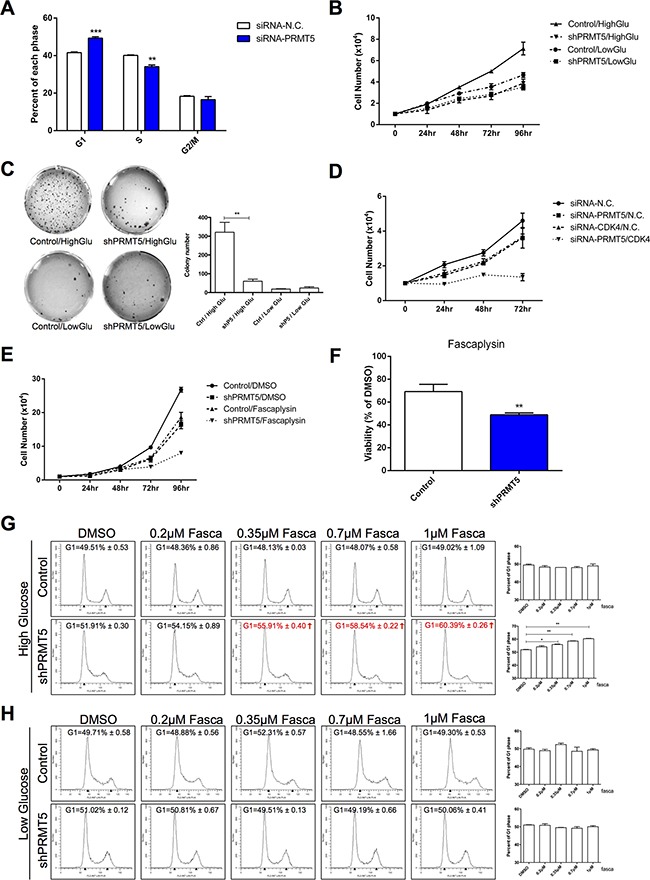
Sensitization of HCC cells to a CDK4 inhibitor by PRMT5 depletion upon glucose induction **A.** HepG2 cells were transfected with siRNA-PRMT5 and siRNA-N.C. as a control for 48 h. Cells were analyzed by FACS, and percentages of cells in each phase were determined by using Modfit LT software. Data are presented as the mean ± SEM and tested with t-test from three independent experiments, **P < 0.01, ***P < 0.001. **B.** Control and shPRMT5 HuH-7 cells were cultured in high (4.5g/L) and low (1g/L) glucose DMEM media, and the cell numbers were counted every 24 h. **C.** Control and shPRMT5 HuH-7 cells were seeded and cultured in high or low glucose medium with agarose gel to perform colony formation assay. The pictures of crystal violet staining cells were presented on the left, and the colony numbers were calculated on the right and tested with t-test. **P < 0.01. **D.** Numbers of HepG2 cells transfected with siRNA-N.C./PRMT5/CDK4 were counted every 24 h. **E.** Numbers of control and shPRMT5 HepG2 cells treated with 1 μM fascaplysin (or DMSO as control) were counted every 24 h. **F.** Cell viability with fascaplysin was determined at 96 h. **G**, **H.** Control and shPRMT5 HepG2 cells cultured in high or low glucose were treated with 0.2 μM, 0.35 μM, 0.7 μM or 1 μM fascaplysin (DMSO as control) for 2 h. Cells were analyzed by FACS, then percentages of cells in the G1 phase were determined by using Modfit LT software and tested with t-test. *P < 0.05, **P < 0.01.

Since PRMT5 is essential for tumor cell cycle and proliferation, we investigated the role of PRMT5 in HCC cell cycle regulation by flow cytometry. As shown in Figure 2A, a significant increase in the proportion of HepG2 cells with PRMT5 knocked down by siRNA were in the G1 phase, while and those in the S phase were decreased (similar results in PRMT5 knockdown by shRNA in HuH-7 cells, Supplementary Figure S2). Tumor cells need massive glucose uptake, and previous studies have shown that PRMT5 stimulates hepatic glucose metabolism [22, 23]. Therefore, we examined whether PRMT5 promotes HCC cell proliferation by relying on glucose induction. Compared with the control HuH-7 cells, PRMT5 knockdown by shRNA obviously suppressed the proliferation of HuH-7 cells in the high glucose condition (Figure 2B and 2C). However, in the low glucose condition, both the control-shRNA and PRMT5-shRNA HuH-7 cell groups presented slower growth than in those in the high glucose condition (Figure 2B and 2C). These results indicated that PRMT5 promoted HCC cell proliferation under glucose induction.

### PRMT5 depletion sensitizes HCC cells to CDK4 inhibitor upon glucose induction

In the high glucose condition, HepG2 cells with both PRMT5 and CDK4 knocked down by siRNA showed slower growth than those with only PRMT5 or CDK4 interference (Figure 2D). Moreover, using a small molecule CDK4 inhibitor fascaplysin, we also observed a more repressive effect in shPRMT5 HepG2 cells than control cells (Figure 2E). We also compared the effect of fascaplysin on cell viability of control or shPRMT5 HepG2 cells and observed that the PRMT5 knockdown cells had a lower cell survival rate with fascaplysin (Figure 2F). Furthermore, to examine the effects of PRMT5 and CDK4 in cell cycle regulation, control and shPRMT5 HepG2 cells cultured in high glucose were exposed to different concentrations of fascaplysin for 2 h. With the increase of fascaplysin concentration, a visible increase of shPRMT5 treated HepG2 cells in the G1 phase was observed, while control cells showed no such increase (Figure 2G). However, in low glucose, no noticeable change in either control or shPRMT5 treated cells in the G1 phase was observed in the 2-h treatment (Figure 2H). Thus, our results suggested that PRMT5 and CDK4 promoted HCC cell proliferation and cell cycle progression upon glucose induction, and PRMT5 depletion sensitized HCC cells to the CDK4 inhibitor.

### PRMT5 activates CDK4-pRB-E2F-mediated transcription in high glucose condition

**Figure 3:**
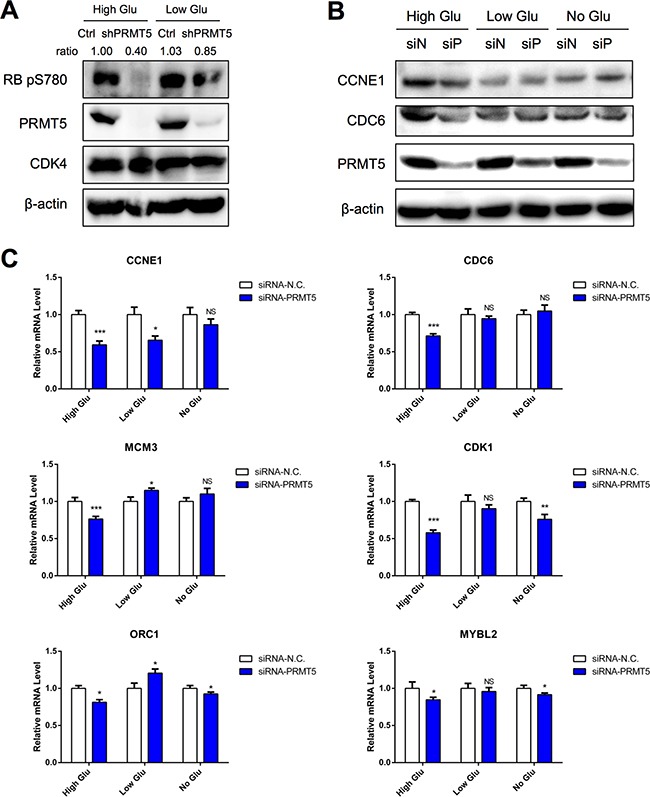
PRMT5 activation of CDK4-pRB-E2F-mediated transcription in high glucose condition **A.** Phosphorylation levels of RB serine780 in control or shPRMT5 HepG2 cells cultured with high/low glucose for 24 h were examined by Western blot. **B.** HepG2 cells were transfected with siRNA-PRMT5 (or siRNA-N.C. as control) for 24 h and then cultured in no/low/high glucose media for 48 h. Protein levels of CCNE1 and CDC6 were examined by Western blot. **C.** HepG2 cells were transfected with siRNA-PRMT5 (or control siRNA) for 24 h and then cultured in no/low/high glucose media for 24 h. The mRNA levels of target genes were examined by qRT-PCR. Data are presented as the mean ± SD and tested with t-test from three independent experiments. NS (no significant difference), *P < 0.05, **P < 0.01, ***P < 0.001.

Next, we examined the role of PRMT5 on CDK4 function. In the high glucose condition, PRMT5 knockdown HepG2 cells displayed a weaker phosphorylation of RB serine780 (Figure 3A); however, it was not significantly changed in the low glucose condition (Figure 3A). Since RB serine780 phosphorylation activates the CDK4-RB-E2F pathway, we examined whether PRMT5 would affect its function. Using Western blot assay and quantitative real-time PCR (qRT-PCR), we analyzed the protein and mRNA levels of RB-E2F downstream genes between control and PRMT5-siRNA treated HepG2 cells in various glucose conditions. The results indicated that the protein levels of CCNE1 and CDC6 in high glucose-cultured siPRMT5 cells were decreased (Figure 3B). And the mRNA levels of most of these downstream genes (*CCNE1, CDC6, MCM3, CDK1, MYBL2, ORC1*) were more significantly down-regulated in siPRMT5 treated cells cultured in high glucose medium (Figure 3C). Thus, our results demonstrated that PRMT5 promoted cell cycle progression through CDK4-RB-E2F transcriptional activation in the high glucose condition.

### PRMT5 interacts with CDK4

**Figure 4:**
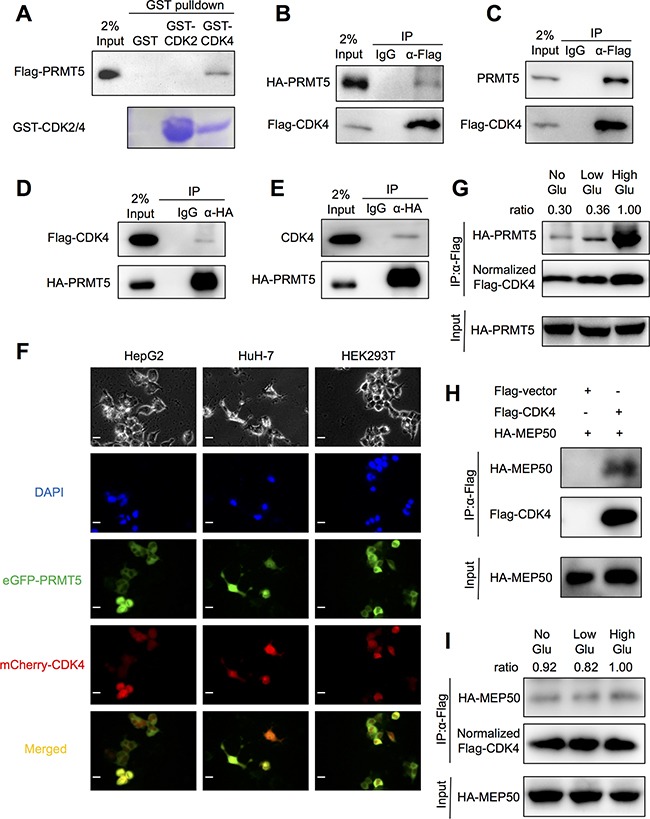
PRMT5 interaction with CDK4 **A.** GST-pull down assays between GST-fused CDK2/4 (or GST alone as control) and Flag-PRMT5 overexpressed in HKT293T cell lysates were performed. **B, C, D, E.** HEK293T cells were transfected with Flag-CDK4 or HA-PRMT5 (or both) for 48 h. The interaction between exogenous (or endogenous) PRMT5 and CDK4 was examined by co-immunoprecipitation assays followed by Western blot. **F.** HepG2, HuH-7 and HEK293T cells were co-transfected with eGFP-PRMT5 and mCherry-CDK4 for 48 h. The eGFP (green) and mCherry (red) signals were acquired by a fluorescence microscope at 40× magnification (Scale bar, 10 μm). **G.** HepG2 cells were co-transfected with HA-PRMT5 and Flag-CDK4 for 24 h in normal conditions and then cultured in no/low/high glucose media for 24h. The interaction was examined by co-immunoprecipitation assays followed by Western blot. **H.** HEK293T cells were transfected with Flag-CDK4 (or empty vector) and HA-MEP50 for 48 h. The interaction between exogenous MEP50 and CDK4 was examined by co-immunoprecipitation assays followed by Western blot. **I.** HepG2 cells were co-transfected with HA-MEP50 and Flag-CDK4 for 24 h in normal conditions and then cultured in no/low/high glucose media for 24h. The interaction was examined by co-IP assays followed by Western blot.

In a previous study, CDK4-CCND1 interaction was shown to activate PRMT5 methyltransferase through the regulatory factor MEP50 [21]. However, our study suggests an interaction exists between PRMT5 and CDK4. This interaction was confirmed using a GST-fused CDK4 recombinant protein to pull down purified Flag-tagged PRMT5. As a negative control, another cell cycle regulator CDK2 which could not interact with PRMT5 was used (Figure 4A). Moreover, exogenous HA-tagged PRMT5 and endogenous PRMT5 expressed in HEK293T cells could be co-immunoprecipitated by purified Flag-tagged CDK4 (Figure 4B and 4C). Conversely, exogenous Flag-tagged CDK4 and endogenous CDK4 could be immunoprecipitated by purified HA-tagged PRMT5 (Figure 4D and 4E). As PRMT5 has been reported an interaction with anti-Flag antibody [24, 25], we performed the endogenous co-IP assay (Supplementary Figure S3A) and exogenous Flag-PRMT5 precipitating HA-CDK4 assay (Supplementary Figure S3B), and confirmed PRMT5 could interact with CDK4 in the absence of anti-Flag antibody. Next, HEK293T cells and HCC cell lines HepG2 and HuH-7 were transfected with eGFP-PRMT5 (green) and mCherry-CDK4 (red), and yellow signals indicating their co-localization were detected both in cytoplasm and nucleus in the merged images (Figure 4F). Furthermore, the interaction between PRMT5 and CDK4 was found to be enhanced in HepG2 cells cultured in high glucose (Figure 4G). As PRMT5-MEP50 complex was essential for PRMT5 function, next we tested whether CDK4 could interact with MEP50. Co-IP assays confirmed the interaction between CDK4 and MEP50 (Figure 4H). However, glucose did not significantly influence the interaction between CDK4 and MEP50 (Figure 4I). Together, our results suggested that PRMT5-MEP50 complex interacted with CDK4, and high glucose condition mainly enhanced PRMT5-CDK4 interaction not MEP50-CDK4.

### PRMT5 inhibits interaction between CDK4 and CDKN2A upon glucose induction

**Figure 5:**
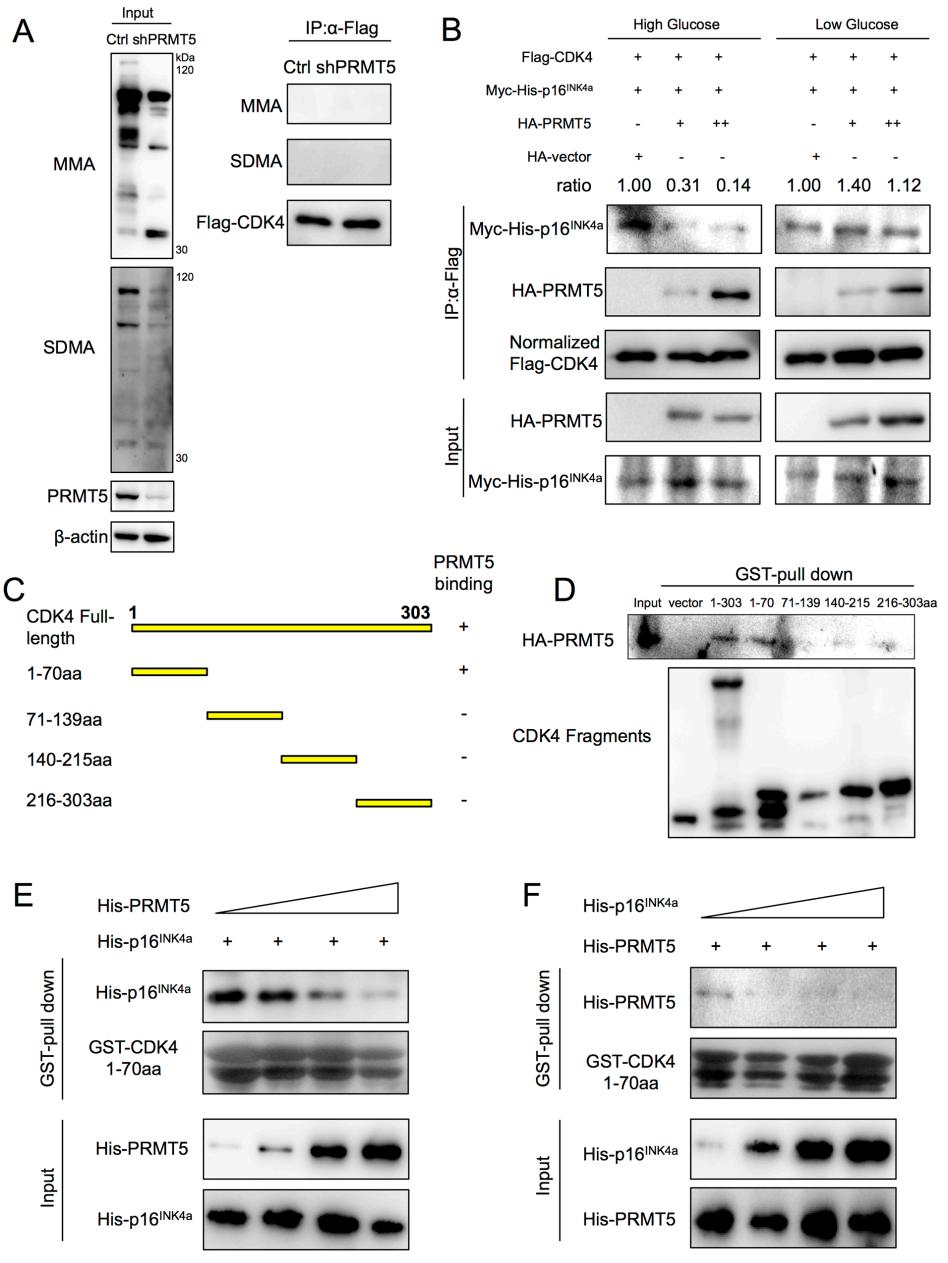
PRMT5 competitive inhibition of interaction between CDK4 and CDKN2A **A.** Flag-CDK4 proteins in Control or shPRMT5 HepG2 cells were immunoprecipitated by anti-Flag M2 affinity gel, and the mono-methylated arginine (MMA) and symmetric dimethylated arginine (SDMA) levels of CDK4 were examined by Western blots. **B.** HEK293T cells were co-transfected with Flag-CDK4, Myc-6×His-p16^INK4a^ and HA-PRMT5 (0.4 μg or 4 μg, with HA-empty vector as control) for 24 h and cultured in high/low glucose media for 24 h. Flag-CDK4 proteins were immunoprecipitated with an anti-Flag M2 affinity gel, and Myc-His-p16^INK4a^ protein levels in the samples were examined by Western blot. **C.** Full-length CDK4 (NP_000066, NCBI) and its fragments used in this study. **D.** GST-pull down assays between GST-fused CDK4 fragments and HA-PRMT5 overexpressed in HEK293T cell lysates were performed. **E**, **F.** Binding reaction (500 μl) containing 100 μl purified His-p16^INK4a^ (or His-PRMT5) and increasing amounts (50 μl, 100 μl, 200 μl, 400 μl) of purified His-PRMT5 (or His-p16^INK4a^) was incubated with GST-fused CDK4 1-70 aa. Protein levels of His-p16^INK4a^ (or His-PRMT5) pulled down were examined by Western blot.

The mechanism of PRMT5 and CDK4 interaction was further explored. Although PRMT5 is a symmetric arginine dimethylase, monomethylation (MMA) and symmetric dimethylation (SDMA) of CDK4 through the interaction were not detected (Figure 5A). Using the methyltransferases inhibitor adenosine-2′, 3′-dialdehyde (Adox, which inhibits SAH hydrolase and accumulates SAH, resulting in inhibiting methyltransferases) (Supplementary Figure S4A) and PRMT5 enzymatic mutation Δmut (Supplementary Figure S4B), we also did not observe any change of MMA or SDMA modification of CDK4. Because CDK4 activation depends on CCND-stimulative binding and CDKN2A-suppressive binding [7, 8], we needed to determine whether PRMT5 would influence CDK4-CCND or CDK4-CDKN2A binding. Thus, we co-transferred Flag-tagged CDK4, Myc-6×His-tagged p16^INK4a^ (the major isoform of CDKN2A) and different quantities (0, 0.4 and 4 μg) of HA-tagged PRMT5 into high/low glucose cultured HEK293T cells, and the cell lysis solution was immunoprecipitated with an anti-Flag M2 antibody. Interestingly, p16^INK4a^ weakly bound to CDK4 in the presence of PRMT5 in the high glucose condition (Figure 5B). However under the low glucose condition, PRMT5 could not affect the interaction between CDK4 and p16^INK4a^ (Figure 5B). Moreover, using the same experimental approach, we observed that CDK4-CCND1 binding was not influenced by PRMT5 (Supplementary Figure S4C). These data suggested that upon glucose induction, PRMT5 inhibited the interaction between CDK4 and CDKN2A.

### PRMT5 and CDKN2A compete for CDK4 binding

To identify sequence motifs of CDK4 required for PRMT5 binding, we divided the CDK4 protein into four sections according to its secondary structure (Uniprot database): amino acids 1-70, 71-139, 140-215 and 216-303 (Figure 5C). GST alone and GST fusion proteins to CDK4 fragments were produced in *Escherichia coli* BL21 and then purified (Supplementary Figure S4D). GST-pull down assays showed the ability of HA-PRMT5 to interact with CDK4 full-length (1-303 aa) and CDK4 1-70 aa proteins (Figure 5D), which includes the ATP-binding domain, cyclin-binding domain and some inhibitory sites [8]. Since PRMT5 was needed for CDK4 1-70 aa binding and inhibited the binding between CDK4 and CDKN2A, we next examined whether PRMT5 and CDKN2A competitively interact with CDK4 1-70 aa. We purified 6× His-tagged PRMT5 and p16^INK4a^ *in vitro* and then incubated GST-fused CDK4 1-70 aa with His-tagged p16^INK4a^ and increasing concentrations of His-tagged PRMT5. As the levels of PRMT5 were increased, the protein levels of p16^INK4a^ pulled down by CDK4 1-70 aa decreased (Figure 5E). Similarly, the protein levels of PRMT5 pulled down by CDK4 1-70 aa also decreased as the levels of p16^INK4a^ were increased (Figure 5F). These results suggested that PRMT5 and CDKN2A competed for binding with CDK4 in the 1-70 aa region.

### PRMT5-binding mutant CDK4 R24A inhibits HCC cell cycle progression

**Figure 6:**
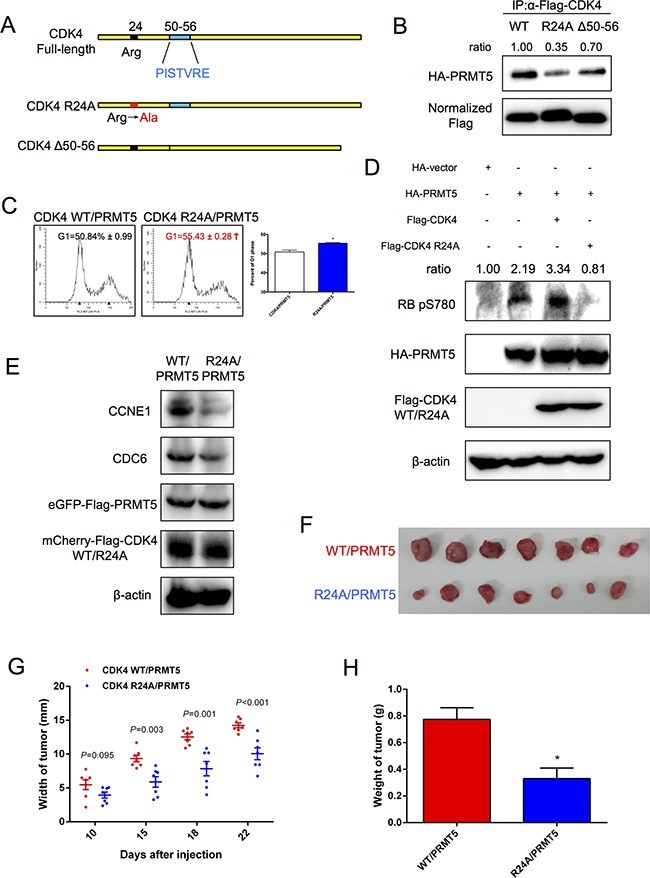
CDK4 mutant R24A inhibition of HCC cell cycle progression **A.** Structure of CDK4 mutants used in this study. **B.** HEK293T cells were co-transfected with Flag-CDK4 WT (R24A or Δ50-56) and HA-PRMT5 for 48 h. The interaction was examined by co-immunoprecipitation assays followed by Western blot. **C.** HepG2 cells stably expressing CDK4 WT/PRMT5 and CDK4 R24A/PRMT5 were analyzed by FACS, and then percentages of cells in the G1 phase were determined using Modfit LT software and tested with t-test. *P < 0.05. **D.** HepG2 cells were transfected with HA-empty vector, HA-PRMT5, HA-PRMT5/Flag-CDK4 or HA-PRMT5/Flag-CDK4 R24A for 48 h. The phosphorylation level of RB serine780 was examined by Western blot. **E.** Protein levels of CCNE1 and CDC6 in HepG2 cells stably expressing CDK4 WT/PRMT5 and CDK4 R24A/PRMT5 were examined by Western blot. **F.** Nude mice were injected with 5 × 10^6^ HepG2 cells stably expressing CDK4 WT/PRMT5 (upper row) and CDK4 R24A/PRMT5 (lower row). Tumors isolated from mice were imaged. **G.** Average widths of tumors were recorded on various days after injection, and *P* < 0.05 was considered significant. **H.** Weights of tumors were recorded when the mice were sacrificed. Data are presented as the mean ± SEM and tested with t-test, *P < 0.05.

Previous studies have shown that the arginine 24 residue within the CDK4 1-70 amino acid region is essential for CDKN2A-binding [26], and mutations R24C and R24H of CDK4 were shown to inhibit protein binding with CDKN2A in melanoma [27–29]. Here, we constructed the Flag-tagged CDK4 R24A mutant (Figure 6A) and validated that R24A had a lower level of binding with p16^INK4a^ than the wild-type (WT) protein (Supplementary Figure S5A). Interestingly, the interaction between PRMT5 and CDK4 R24A was also diminished (Figure 6B). However, the CCND1-misbinding mutant CDK4 Δ50-56, lacking the 50-56 aa sequence PISTVRE (Figure 6A), still maintained the majority of the interaction with PRMT5 (Figure 6B). To determine whether R24A could function in the presence of PRMT5, cell growth and cell cycle progression in HuH-7 and HepG2 cells were analyzed. As shown in Supplementary Figure S5B and S5C, compared to CDK4/PRMT5, R24A/PRMT5 significantly inhibited HCC cell proliferation. The flow cytometry analysis also showed that R24A/PRMT5 increased the percentage of cells in the G1 phase relative to CDK4/PRMT5 (Figure 6C). Furthermore, we investigated whether CDK4 R24A also affected the CDK4-RB-E2F pathway. The phosphorylation level of RB serine780 was decreased in HepG2 cells expressing R24A/PRMT5 (Figure 6D). Similarly, R24A/PRMT5 caused down-regulation of the mRNA (Supplementary Figure S5D) and protein (Figure 6E) levels of RB-E2F downstream genes in comparison to CDK4/PRMT5. Taken together, these results suggested that CDK4 arginine24 was indispensable for PRMT5 function, and the CDK4 R24A mutant inhibited HCC cell growth and cell cycle progression.

### CDK4 R24A inhibits HCC tumor growth

To determine whether the CDK4 mutant R24A also would inhibit HCC growth *in vivo*, we conducted xenograft tumor studies. Nude mice were injected with 5 × 10^6^ HepG2 cells stably expressing CDK4 WT and PRMT5 (or stably expressed CDK4 R24A and PRMT5). The consistent expression levels of PRMT5 and CDK4 (WT and R24A) in this two cell lines were validated (Figure 6E). As shown in Figures 6F and S5E, tumors in mice injected with CDK4 WT/PRMT5 HepG2 cells were larger than those in mice injected with CDK4 R24A/PRMT5 HepG2 cells. Furthermore, injection of HepG2 cells transfected with CDK4 WT/PRMT5 also resulted in a larger tumor width (Figure 6G) and tumor weight (Figure 6H) than those with CDK4 R24A/PRMT5. Our results suggested that the weak binding of CDK4 R24A with PRMT5 inhibited HCC tumor growth.

## DISCUSSION

PRMT5 and CDK4 are highly expressed in HCC. In our study, we demonstrated the glucose-induced facilitation of HCC cell growth by PRMT5, which can promote the G1/S cell cycle transition. Moreover, cell proliferation assays showed that shPRMT5 HepG2 cells were more sensitive to fascaplysin, and the FACS analysis suggested higher fascaplysin sensitivity for PRMT5-depleted cells in the G1/S transition under the high glucose condition. In other words, under glucose induction, PRMT5-depleted HCC cells showed CDK4 instability and high CDK4 inhibitor sensitivity. On the other hand, PRMT5 was found to interact with and affect the function of CDK4. With high glucose, PRMT5 competitively inhibited the interaction between CDK4 and CDKN2A, leading to CDK4-RB-E2F transcriptional activation. Furthermore, relative to the coexpression of CDK4 WT with PRMT5, that of the CDK4 mutant R24A with PRMT5 resulted in the arrest of HCC cell cycle and proliferation. In conclusion, the interaction between PRMT5 and CDK4 rendered a tumor-promoting effect.

The relationship between PRMT5 and CDKs in previous studies have been identified in that PRMT5 plays a role in regulating symmetric dimethylation of histones through the interaction of methylosome protein 50 (MEP50) with CDK4 [21], CDK8 or CDK19 [30]. For example, the previous works have shown that CCND1/CDK4 phosphorylates the PRMT5 co-factor MEP50 and activates PRMT5 methyltransferase in CUL4 transcriptional suppression. Those observations suggest a critical oncogenic role of PRMT5 and CDK4 in tumorigenesis. Here, we provide considerable evidence of a positive role for PRMT5 and CDK4 in tumor cell cycle regulation. Our study clearly described the non-methyltransferase activity of PRMT5 by competitively preventing CDK4 from binding CDKN2A, thereby increasing the CDK4-RB-E2F transcriptional activity. Although we affirmed MEP50 interacted with CDK4, the interaction could not be significantly induced by glucose. That means glucose may promote the formation of PRMT5/MEP50-CDK4 complex, however, it mainly enhances the link of PRMT5-CDK4 not MEP50-CDK4 in a molecular level. Thus, our results support a supplemental function for CDK4-PRMT5 transcriptional repression in contributing to tumor growth.

Commonly, PRMT5 contributes to tumorigenesis and cell cycle imbalance as an arginine methyltransferase. PRMT5 methylates arginine residues of histones or non-histones leading to transcriptional silencing, including the cell cycle regulators. For example, acting as a tumor suppressor, E2F1 methylated by PRMT5 can increase tumor cell growth and inhibit apoptosis [31]. However, E2F1 plays an accelerative role in cell cycle regulation of tumor growth, which is dependent on pRB and CDKs. Here, we provide evidence for PRMT5 functioning in a non-enzymatic manner. The competitive binding of CDK4-CDKN2A or PRMT5-CDK4 is alternatively decided by glucose induction. Thus, it turns the pRB-E2F1 pathway into a transcriptionally activated state or depressed state. The results suggest a crucial mechanism for PRMT5 in cell cycle regulation contributing to oncogenesis.

There are varieties of factors that affect cell proliferation, in which an important one is regulation of cell cycle progression. Cell cycle activators promote cell cycle progression, the ratio of apoptosis cells decrease and then cells grow faster. This is a cascade amplification process. In our study, PRMT5 as an upstream regulator promoted cell cycle through CDK4 in performance at G1-S transition. We observe a limited change of the ratio of G1 and S phase when PRMT5 was short-term knockdown, but over time the effect of cell proliferation could present a large variation. On the other hand, PRMT5 can also promote cell growth in other ways, such as accelerating cell metabolites biosynthesis [32], and cell cycle regulation is not the only pathway. Therefore, this is why cell cycle effect looks not dramatic but proliferation effect looks significant.

As a major nutrient, glucose is critically required for cellular biosynthesis and energy supply. Compared with normal cells, tumor cells metabolize more glucose to lactate via glycolysis, a phenomenon called the Warburg effect [33]. Due to the liver acting as the main metabolic organ, HCC is an aggressive cancer requiring high glucose uptake. In our study, PRMT5 was shown to facilitate HCC cell growth induced by high-level glucose, similar to results of numerous prior studies on PRMT5 function. Despite the oncogenic role of PRMT5, the underlying mechanisms of PRMT5 in HCC are not fully understood. Surprisingly, in our study, PRMT5 directly bound to CDK4 in rapidly growing cells such as HEK293T and HepG2 cells. Interestingly, this whole system is glucose-driven. Glucose has been considered an actuator for the cell cycle, and a high level of it controls the cell cycle progression by increasing CCNA, CCNB1 and CCND1 [34]. In our study, we found that the combination of PRMT5 and CDK4 presented a larger impact on HCC cell cycle progression under the high glucose condition. On the other hand, CDK4 can maintain intercellular glucose homeostasis by controlling glucose and lipid metabolism [35] or repressing gluconeogenesis in a CCND1-dependent manner [36]. Taken together, results of previous studies and our current work have revealed PRMT5 and CDK4 as a crucial link for cell cycle and glucose metabolism regulation.

Since results of the GST-pull down and co-immunoprecipitation assay suggested an interaction between PRMT5 and CDK4, we predicted that PRMT5 impacts HCC cell proliferation via the effect of binding with CDK4. CDK4 is well known as an oncogene and a significant clinical marker in HCC progression [37]. Moreover, some CDK4-specific inhibitors have been applied in research and clinical trials. Fascaplysin is a marine bis-indole alkaloid and shows specific CDK4 inhibitory activity with an IC50 of 0.35 μM, while its activity was observed to be low against other CDKs [38]. Another CDK4 inhibitor PD0332991, which can significantly suppress proliferation of hepatoma cells, has been evaluated in a clinical phase II study of liposarcomas [39, 40]. In this study, effects of fascaplysin on HCC cell growth and G1/S transition arrest were more significant in the absence of PRMT5. The results imply a more efficient therapeutic strategy for CDK4 inhibitors in the treatment of HCC.

In addition, CDK4 mutations have been reported in several types of cancer. The phosphorylation of CDK4 at threonine172 was found to be essential for CDK4-CCND activating binding, while the CDK4 T172A mutant displayed very weak activity [41, 42]. CDK4 R24C and R24H were found in human melanoma as tumor activating mutations, but they showed no effect on sporadic pituitary adenomas, insulinomas or Leydig cell tumors [43]. In this study, our data showed an important function of the CDK4 mutant R24A in HCC cells. In comparison to WT CDK4, the R24A mutant did not influence the change in G1 phase in HCC cells (data not shown). However, in the presence of PRMT5, R24A significantly arrested G1/S transition and HCC cell growth. Although R24A bound less strongly to CDKN2A, the weak interaction between PRMT5 and R24A showed more evidently that CDK4 R24A suppresses HCC cell cycle progression in a PRMT5-dependent manner. Thus, an important mechanism was discovered for the roles of PRMT5 and CDK4 in HCC.

**Figure 7:**
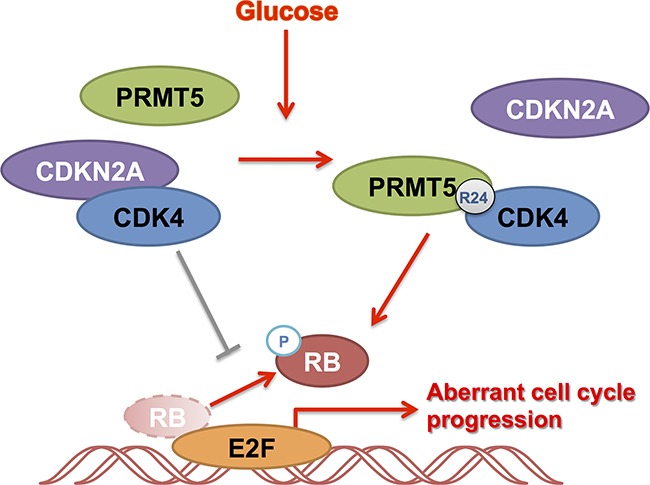
Summary of the role of PRMT5 and CDK4 in HCC cell cycle regulation CDK4 binding with CDKN2A inhibited HCC cell cycle. Under glucose induction, PRMT5 was shown to emphatically interact with CDK4, releasing it from CDKN2A and leading the CDK4-RB-E2F axis to an active state for tumor growth.

In summary, we have demonstrated that PRMT5 is a conditional regulator of CDK4 in HCC. In the high glucose condition, PRMT5 was shown to emphatically interact with CDK4, releasing it from CDKN2A and leading the CDK4-RB-E2F axis to an active state for tumor growth (Figure 7). Our study may provide a strategy for improving CDK4-targeting treatments against HCC.

## MATERIALS AND METHODS

### Cell culture, plasmids and reagents

All cell lines were purchased through ATCC and cultured in DMEM (0/1/4.5 g/l D-glucose, GIBCO) supplemented with 10% FBS (GIBCO), 100 mg/ml penicillin and 100 mg/ml streptomycin sulfate (GIBCO) at 37°C and 5% CO_2_. Stable PRMT5 knockdown and control cells were cultured in the same media with the addition of 1 μg/ml puromycin (Life Technologies). PCR-amplified human PRMT5, CDK4 and CDK4 mutations were cloned into pcDNA4TO-Flag/HA vectors. Human CCND1 and p16^INK4a^ were cloned into the pcDNA3.0-Myc-6×His vector. Prokaryotic expression plasmids pEGX-4T1 and pET-28a were also used. All plasmids were verified by DNA sequencing. Lipofectamine 2000 (Invitrogen) for transfection and fascaplysin (Santa Cruz Biotechnology) were purchased.

### Immunohistochemistry

All experimental procedures using human tissues were approved by the Human Ethics Committee of Institute of Health Sciences. The samples were obtained by surgical resection from patients who gave consent at Renji Hospital. The tissue sections were treated with 3% H_2_O_2_ to block endogenous peroxidase activity and incubated with primary antibodies (anti-CDK4 at 1:2000 and anti-PRMT5 at 1:2000). The sections were then incubated with an HRP-conjugated secondary antibody, followed by treatment with diaminobenzidine chromogen to visualize the signals. Staining of CDK4 and PRMT5 was scored by two independent researchers based on the distribution and intensity of signals. The distribution (percentage of stained tissue area) was scored as 0 (0–5%), 1 (5–25%), 2 (25–50%), 3 (50–75%), 4 (75–100%). The intensity of staining was scored as 0 (no staining), 1 (weak), 2 (moderate) and 3 (strong). The final scores of CDK4 or PRMT5 expression in HCC and adjacent tissues were on a scale of 0-12. Samples were defined as having high expression (score > 6) or low expression (score ≤ 6). Pearson's test was applied to determine the correlation between CDK4 and PRMT5 using SPSS 20 software.

### Immunoblotting and antibodies

Cells were lysed in RIPA buffer containing protease inhibitors for 30 min on ice, followed by centrifugation at 15000 × *g* for 30 min. After SDS-PAGE, extracted proteins were transferred onto PVDF membranes (GE Healthcare) and probed with primary antibodies at 4°C overnight. The membranes were then incubated with secondary antibodies for 1 h, and the bands were captured through an ECL HRP substrate (Millipore). Anti-PRMT5 (Abcam, ab109451), anti-CDK4 (Santa Cruz Biotechnology, sc-260), anti-β-actin (Cell Signaling Technology, #4967), anti-β-tubulin (Cell Signaling Technology, #2146), anti-Flag M2 (Sigma-Aldrich, F3165), anti-HA (Biolegend, 901503), anti-6× His (Proteintech, 66005-1-Ig), anti-pRB S780 (Abcam, ab47763), anti-Mono-Methyl Arginine (Cell Signaling Technology, #8015) and anti-SDMA (Millipore, 07-413), anti-CCNE1 (Proteintech, 11554-1-AP), anti-CDC6 (Proteintech, 11640-1-AP) antibodies were purchased.

### shRNA

HepG2 and HuH-7 cells were infected by lentivirus with Control shRNA (sense 5′- GATCCTTCTCCGAACGTGTCACGTTCAAGAGACGTGACACGTTCGGAGAATTTTTTG-3′, antisense 5′-AATTCAAAAAATTCTCCGAACGTGTCACGTCTCTTGAACGTGACACGTTCGGAGAAG-3′) and shPRMT5 (sense 5′-GATCCGGGACTGGAATACGCTAATTTCAAGAGAATTAGCGTATTCCAGTCCCTTTTTTG-3′, antisense 5′-AATTCAAAAAAGGGACTGGAATACGCTAATTCTCTTGAAATTAGCGTATTCCAGTCCCG-3′). Then the cells were screened by puromysin.

### Transfection of siRNA

Cells were transfected with oligo small silencing RNAs using Lipofectamine 2000 in serum-free Opti-MEM (GIBCO, NY, USA) according to the manufacturer's instruction. The sequences of siRNA oligos used in this study are listed as following: *PRMT5* (sense 5′-GGGACUGGAAUACGCUAAUTT-3′, antisense 5′- AUUAGCGUAUUCCAGUCCCTT-3′), *CDK4* (sense 5′-CAGUUCGUGAGGUGGCUUUAC-3′, antisense 5′- GUAAAGCCACCUCACGAACUG-3′), and *Negative Control* (sense 5′-UUCUCCGAGCGUGUCACGUTT-3′, antisense 5′-ACGUGACACGUUCGGAGAATT-3′).

### Quantitative real-time PCR

Total RNA was isolated using a Trizol kit (Omega, GA, USA), and cDNA was synthesized using a cDNA synthesis kit (Takara, Otsu, Japan). Quantitative real-time PCR was performed using the SYBR Green PCR Master Mix (Takara, Otsu, Japan) on the StepOnePlus Real-Time PCR System (Applied Biosystems, USA). The primers used in this study are listed as following: *CCNE1*-Forward Primer (AGATGTAGGCCGGGTGATCT), *CCNE1*-Reverse Primer (CCGCCCTGGATCATGAAGTC), *CCNA2*-F (TGATGTTGGGCAACTCTGCG), *CCNA2*-R (GTGCAACCCGTCTCGTCTTC), *CDK1*-F (AGCCGG GATCTACCATACCC), *CDK1*-R (AGGAACCCCTTC CTCTTCACT), *CDC6*-F (TCATGCCTCAAACCCG ATCC), *CDC6*-R (TGTCATCGCCCAGACGTTTC), *ORC1*-F (GGCAGCAGCTTCGGTTTCTA), *ORC1*-R (TCTTTGGCACCTTCGTGAGG), *MCM3*-F (AGGTAG TTCTTTGGCAGCGG), *MCM3*-R (TCAGAAGCCGG TTAGCCCT), *MYBL2*-F (CTGGTGAGGCAGTTTG GACA), *MYBL2*-R (ACCAGCTCGATGACTTTTTGGT), *E2F1*-F (AAGAGCAAACAAGGCCCGAT), *E2F1*-R (AC AACAGCGGTTCTTGCTCC).

### Cell proliferation assays

HepG2 or HuH-7 cells were plated in 24-well plates with a density of 1 × 10^4^ cells per well. Twenty-four hours after transfection or fascaplysin treatment, cells were harvested every 24 h, and cells numbers were counted using a hemocytometer.

### Colony formation assay

12-well plates were covered with 0.4% agarose gel (Gibco), and then cells in 0.375% agarose gel were overlaid on the top of the 0.4% gel. 200 cells were suspended per well and cultured. After 3 weeks, the cells were stained with 0.1% crystal violet. The picture of each well was photographed and the colony number was calculated.

### Flow cytometry

Trypsin digested cells were washed with 1 × PBS, fixed in cold 70% ethanol for 24 h and then incubated with RNase A (Invitrogen) for 30 min at 37°C and 50 μg/ml propidium iodide (Sigma-Aldrich) for 15 min at 4°C shielded from light. Cells were analyzed with a Gallios (Beckman) fluorescence activated cell sorter.

### Co-immunoprecipitation

Extracts of cells overexpressing Flag- or HA-tagged proteins were incubated with antibodies (or IgG as control, Sigma)-binding Protein A/G (Pierce) or anti-Flag M2 affinity gel (Sigma) for 3 h at 4°C. After being washed three times with IP buffer, the samples were analyzed by Western blot.

### GST-pull-down assay

GST-fused CDK2, CDK4 or CDK4-fragments proteins were expressed in *E. coli* BL21 and purified by glutathione-Sepharose 4B beads (GE Healthcare). Cell lysis solution or His-tagged proteins purified by Ni-affinity resins (GE Healthcare) were mixed with purified GST, GST-CDK2, GST-CDK4 or GST-CDK4-fragments beads for 3 h at 4°C. After being washed five times with IP buffer, the samples were analyzed by Western blot.

### Xenograft tumor studies

All experimental procedures using animals were conducted in accordance with the guidelines provided by the Animal Ethics Committee of the Institute of Health Sciences. HepG2 cells stably transfected with CDK4 WT and PRMT5 (or CDK4 R24A and PRMT5) were subcutaneously inoculated in the left (or in the right side) of 4-week-old male BALB/c SCID mice (Shanghai Laboratory Animal Center). Tumor sizes were monitored and measured for growth over a period of 3 weeks, and the results are presented as the mean ± SEM. At the end of the experiments, mice were sacrificed, and tumor tissues were collected and weighed.

### Statistics analysis

All data were statistically analyzed using Graphpad Prism 6 or SPSS 20 software. Two-tailed t-test was used to analyze the difference between two groups. Pearson's test was applied to determine the correlation between CDK4, PRMT5 and clinicopathological parameters. Data are presented as the mean ± SEM or SD, and *P* < 0.05 was considered significant.

## SUPPLEMENTARY FIGURES



## References

[1] Jemal A, Bray F, Center MM, Ferlay J, Ward E, Forman D (2011). Global cancer statistics. CA Cancer J Clin.

[2] Yang JD, Roberts LR (2010). Hepatocellular carcinoma: A global view. Nat Rev Gastroenterol Hepatol.

[3] Bechmann LP, Hannivoort RA, Gerken G, Hotamisligil GS, Trauner M, Canbay A (2012). The interaction of hepatic lipid and glucose metabolism in liver diseases. J Hepatol.

[4] Badvie S (2000). Hepatocellular carcinoma. Postgrad Med J.

[5] Song T, Dou C, Jia Y, Tu K, Zheng X (2015). TIMP-1 activated carcinoma-associated fibroblasts inhibit tumor apoptosis by activating SDF1/CXCR4 signaling in hepatocellular carcinoma. Oncotarget.

[6] Hanahan D, Weinberg RA (2011). Hallmarks of cancer: the next generation. Cell.

[7] Sheppard KE, McArthur GA (2013). The cell-cycle regulator CDK4: an emerging therapeutic target in melanoma. Clin Cancer Res.

[8] Lim S, Kaldis P (2013). Cdks, cyclins and CKIs: roles beyond cell cycle regulation. Development.

[9] Rickheim DG, Nelsen CJ, Fassett JT, Timchenko NA, Hansen LK, Albrecht JH (2002). Differential regulation of cyclins D1 and D3 in hepatocyte proliferation. Hepatology.

[10] Louis-Brennetot C, Coindre JM, Ferreira C, Perot G, Terrier P, Aurias A (2011). The CDKN2A/CDKN2B/CDK4/CCND1 pathway is pivotal in well-differentiated and dedifferentiated liposarcoma oncogenesis: an analysis of 104 tumors. Genes Chromosomes Cancer.

[11] Min EY, Kim IH, Lee J, Kim EY, Choi YH, Nam TJ (2014). The effects of fucodian on senescence are controlled by the p16INK4a-pRb and p14Arf-p53 pathways in hepatocellular carcinoma and hepatic cell lines. Int J Oncol.

[12] Cen L, Carlson BL, Schroeder MA, Ostrem JL, Kitange GJ, Mladek AC, Fink SR, Decker PA, Wu W, Kim JS, Waldman T, Jenkins RB, Sarkaria JN (2012). p16-Cdk4-Rb axis controls sensitivity to a cyclin-dependent kinase inhibitor PD0332991 in glioblastoma xenograft cells. Neuro Oncol.

[13] Pan W, Cox S, Hoess RH, Grafstrom RH (2001). A cyclin D1/cyclin-dependent kinase 4 binding site within the C domain of the retinoblastoma protein. Cancer Res.

[14] Bertoli C, Skotheim JM, de Bruin RA (2013). Control of cell cycle transcription during G1 and S phases. Nat Rev Mol Cell Biol.

[15] Blais A, Dynlacht BD (2004). Hitting their targets: an emerging picture of E2F and cell cycle control. Curr Opin Genet Dev.

[16] Dimova DK, Dyson NJ (2005). The E2F transcriptional network: old acquaintances with new faces. Oncogene.

[17] Li CG, Nyman JE, Braithwaite AW, Eccles MR (2011). PAX8 promotes tumor cell growth by transcriptionally regulating E2F1 and stabilizing RB protein. Oncogene.

[18] Gu Z, Gao S, Zhang F, Wang Z, Ma W, Davis RE, Wang Z (2012). Protein arginine methyltransferase 5 is essential for growth of lung cancer cells. Biochem J.

[19] Jansson M, Durant ST, Cho EC, Sheahan S, Edelmann M, Kessler B, La Thangue NB (2008). Arginine methylation regulates the p53 response. Nat Cell Biol.

[20] Wei TY, Juan CC, Hisa JY, Su LJ, Lee YC, Chou HY, Chen JM, Wu YC, Chiu SC, Hsu CP, Liu KL, Yu CT (2012). Protein arginine methyltransferase 5 is a potential oncoprotein that upregulates G1 cyclins/cyclin-dependent kinases and the phosphoinositide 3-kinase/AKT signaling cascade. Cancer Sci.

[21] Aggarwal P, Vaites LP, Kim JK, Mellert H, Gurung B, Nakagawa H, Herlyn M, Hua X, Rustgi AK, McMahon SB, Diehl JA (2010). Nuclear cyclin D1/CDK4 kinase regulates CUL4 expression and triggers neoplastic growth via activation of the PRMT5 methyltransferase. Cancer Cell.

[22] Tsai WW, Niessen S, Goebel N, Yates JR, Guccione E, Montminy M (2013). PRMT5 modulates the metabolic response to fasting signals. Proc Natl Acad Sci U S A.

[23] Kanamaluru D, Xiao Z, Fang S, Choi SE, Kim DH, Veenstra TD, Kemper JK (2011). Arginine methylation by PRMT5 at a naturally occurring mutation site is critical for liver metabolic regulation by small heterodimer partner. Mol Cell Biol.

[24] Stopa N, Krebs JE, Shechter D (2015). The PRMT5 arginine methyltransferase: many roles in development, cancer and beyond. Cell Mol Life Sci.

[25] Nishioka K (2003). Methods and tips for the purification of human histone methyltransferases. Methods.

[26] Coleman KG, Wautlet BS, Morrissey D, Mulheron J, Sedman SA, Brinkley P, Price S, Webster KR (1997). Identification of CDK4 sequences involved in cyclin D1 and p16 binding. J Biol Chem.

[27] Gaffal E, Landsberg J, Bald T, Sporleder A, Kohlmeyer J, Tuting T (2011). Neonatal UVB exposure accelerates melanoma growth and enhances distant metastases in Hgf-Cdk4(R24C) C57BL/6 mice. Int J Cancer.

[28] Puntervoll HE, Yang XR, Vetti HH, Bachmann IM, Avril MF, Benfodda M, Catricala C, Dalle S, Duval-Modeste AB, Ghiorzo P, Grammatico P, Harland M, Hayward NK (2013). Melanoma prone families with CDK4 germline mutation: phenotypic profile and associations with MC1R variants. J Med Genet.

[29] Veinalde R, Ozola A, Azarjana K, Molven A, Akslen LA, Donina S, Proboka G, Cema I, Baginskis A, Pjanova D (2013). Analysis of Latvian familial melanoma patients shows novel variants in the noncoding regions of CDKN2A and that the CDK4 mutation R24H is a founder mutation. Melanoma Res.

[30] Tsutsui T, Fukasawa R, Shinmyouzu K, Nakagawa R, Tobe K, Tanaka A, Ohkuma Y (2013). Mediator complex recruits epigenetic regulators via its two cyclin-dependent kinase subunits to repress transcription of immune response genes. J Biol Chem.

[31] Cho EC, Zheng S, Munro S, Liu G, Carr SM, Moehlenbrink J, Lu YC, Stimson L, Khan O, Konietzny R, McGouran J, Coutts AS, Kessler B (2012). Arginine methylation controls growth regulation by E2F-1. EMBO J.

[32] Liu L, Zhao X, Zhao L, Li J, Yang H, Zhu Z, Liu J, Huang G (2016). Arginine Methylation of SREBP1a via PRMT5 Promotes De Novo Lipogenesis and Tumor Growth. Cancer Res.

[33] Koppenol WH, Bounds PL, Dang CV (2011). Otto Warburg's contributions to current concepts of cancer metabolism. Nat Rev Cancer.

[34] Grabiec K, Gajewska M, Milewska M, Blaszczyk M, Grzelkowska-Kowalczyk K (2014). The influence of high glucose and high insulin on mechanisms controlling cell cycle progression and arrest in mouse C2C12 myoblasts: the comparison with IGF-I effect. J Endocrinol Invest.

[35] Lee Y, Dominy JE, Choi YJ, Jurczak M, Tolliday N, Camporez JP, Chim H, Lim JH, Ruan HB, Yang X, Vazquez F, Sicinski P, Shulman GI (2014). Cyclin D1-Cdk4 controls glucose metabolism independently of cell cycle progression. Nature.

[36] Bhalla K, Liu WJ, Thompson K, Anders L, Devarakonda S, Dewi R, Buckley S, Hwang BJ, Polster B, Dorsey SG, Sun Y, Sicinski P, Girnun GD (2014). Cyclin D1 represses gluconeogenesis via inhibition of the transcriptional coactivator PGC1alpha. Diabetes.

[37] Lu JW, Lin YM, Chang JG, Yeh KT, Chen RM, Tsai JJ, Su WW, Hu RM (2013). Clinical implications of deregulated CDK4 and Cyclin D1 expression in patients with human hepatocellular carcinoma. Med Oncol.

[38] Hamilton G (2014). Cytotoxic effects of fascaplysin against small cell lung cancer cell lines. Mar Drugs.

[39] Rivadeneira DB, Mayhew CN, Thangavel C, Sotillo E, Reed CA, Grana X, Knudsen ES (2010). Proliferative suppression by CDK4/6 inhibition: complex function of the retinoblastoma pathway in liver tissue and hepatoma cells. Gastroenterology.

[40] Dickson MA, Tap WD, Keohan ML, D'Angelo SP, Gounder MM, Antonescu CR, Landa J, Qin LX, Rathbone DD, Condy MM, Ustoyev Y, Crago AM, Singer S (2013). Phase II trial of the CDK4 inhibitor PD0332991 in patients with advanced CDK4-amplified well-differentiated or dedifferentiated liposarcoma. J Clin Oncol.

[41] Bockstaele L, Kooken H, Libert F, Paternot S, Dumont JE, de Launoit Y, Roger PP, Coulonval K (2006). Regulated activating Thr172 phosphorylation of cyclin-dependent kinase 4(CDK4): its relationship with cyclins and CDK “inhibitors”. Mol Cell Biol.

[42] Bisteau X, Paternot S, Colleoni B, Ecker K, Coulonval K, De Groote P, Declercq W, Hengst L, Roger PP (2013). CDK4 T172 phosphorylation is central in a CDK7-dependent bidirectional CDK4/CDK2 interplay mediated by p21 phosphorylation at the restriction point. PLoS Genet.

[43] Vax VV, Bibi R, Diaz-Cano S, Gueorguiev M, Kola B, Borboli N, Bressac-de Paillerets B, Walker GJ, Dedov, Grossman AB, Korbonits M (2003). Activating point mutations in cyclin-dependent kinase 4 are not seen in sporadic pituitary adenomas, insulinomas or Leydig cell tumours. J Endocrinol.

