# Supplementary material for: PRMT5 competitively binds to CDK4 to promote G1-S transition upon glucose induction in hepatocellular carcinoma
Source: Oncotarget. 2016 Sep 30;7(44):72131–47. doi: 10.18632/oncotarget.12351 (PMC5342150; doi:10.18632/oncotarget.12351)
Supplement: Supplementary file 1 [file oncotarget-07-72131-s001.pdf]

PRMT5 competitively binds to CDK4 to promote G1-S transition upon glucose induction in hepatocellular carcinoma

SUPPLEMENTARY FIGURES

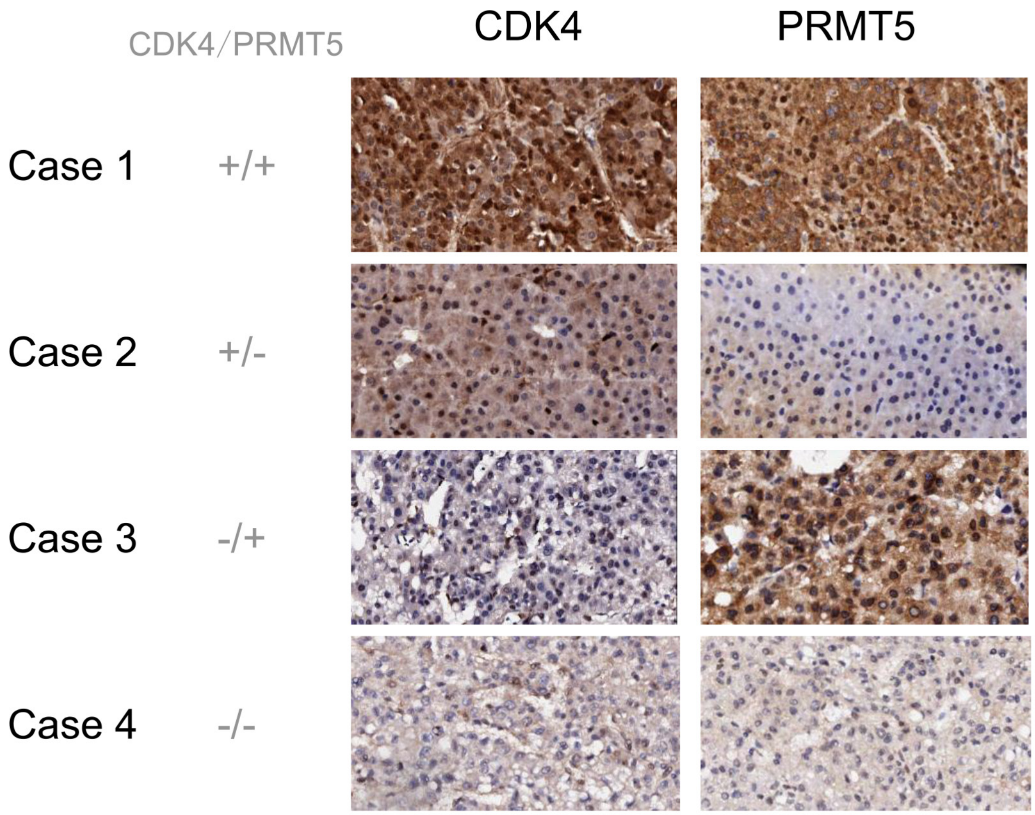

Supplementary Figure S1: Representative histopathologic sections of human HCC for co-expression levels of CDK4/PRMT5. (+) high expression, (-) low expression.

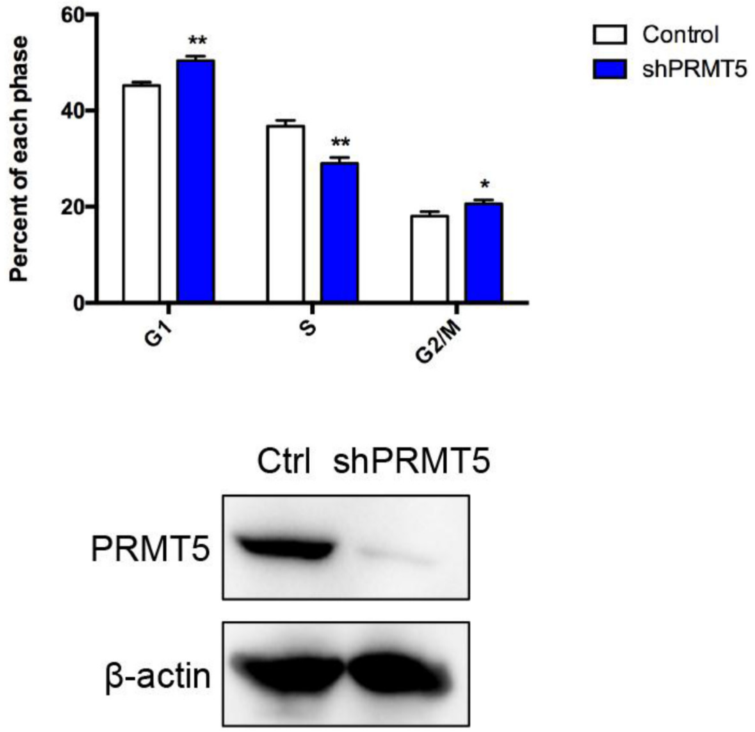

Supplementary Figure S2: Glucose-induced PRMT5 promotes HCC cell proliferation. Control and shPRMT5 HuH-7 cells were analyzed by FACS, and percentages of cells in each phase were determined by using Modfit LT software. Data are presented as the mean ± SEM and tested with t-test from three independent experiments, *P < 0.05, **P < 0.01. The knockdown efficiency of PRMT5-shRNA in HuH-7 cells was validated.

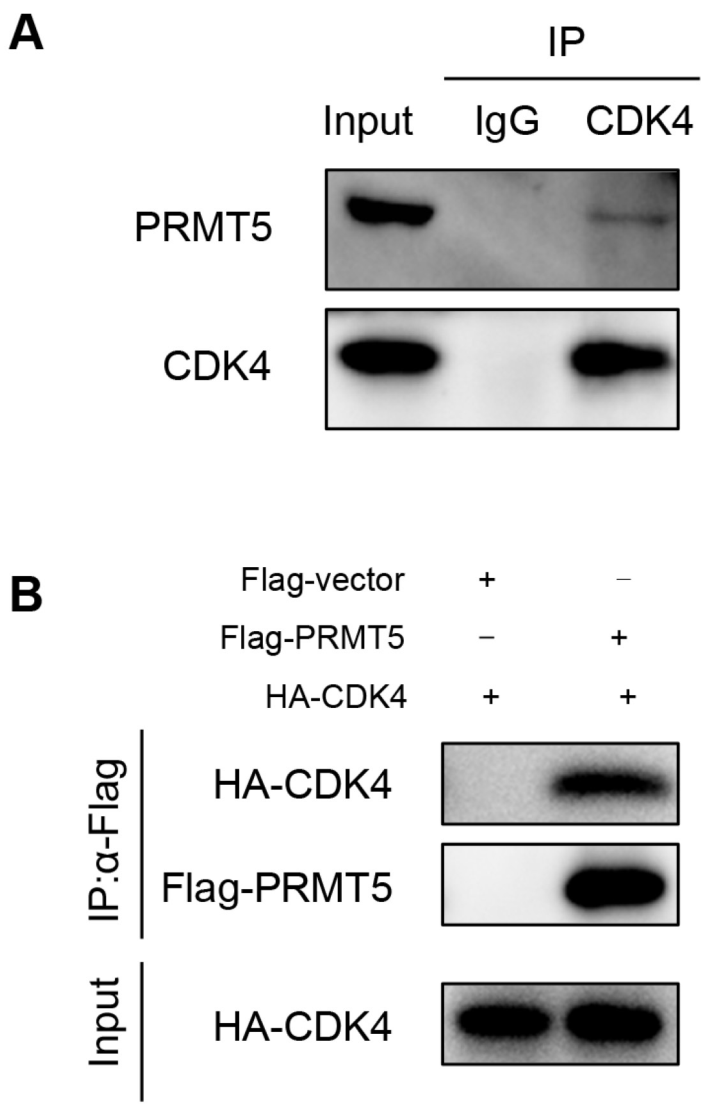

Supplementary Figure S3: PRMT5 interaction with CDK4. A. The interaction between endogenous PRMT5 and CDK4 in HepG2 cells was examined by co-immunoprecipitation assays followed by Western blot. **B.** HEK293T cells were transfected with Flag-PRMT5 (or empty vector) and HA-CDK4 for 48 h. Then co-immunoprecipitation assays followed by Western blot were performed.

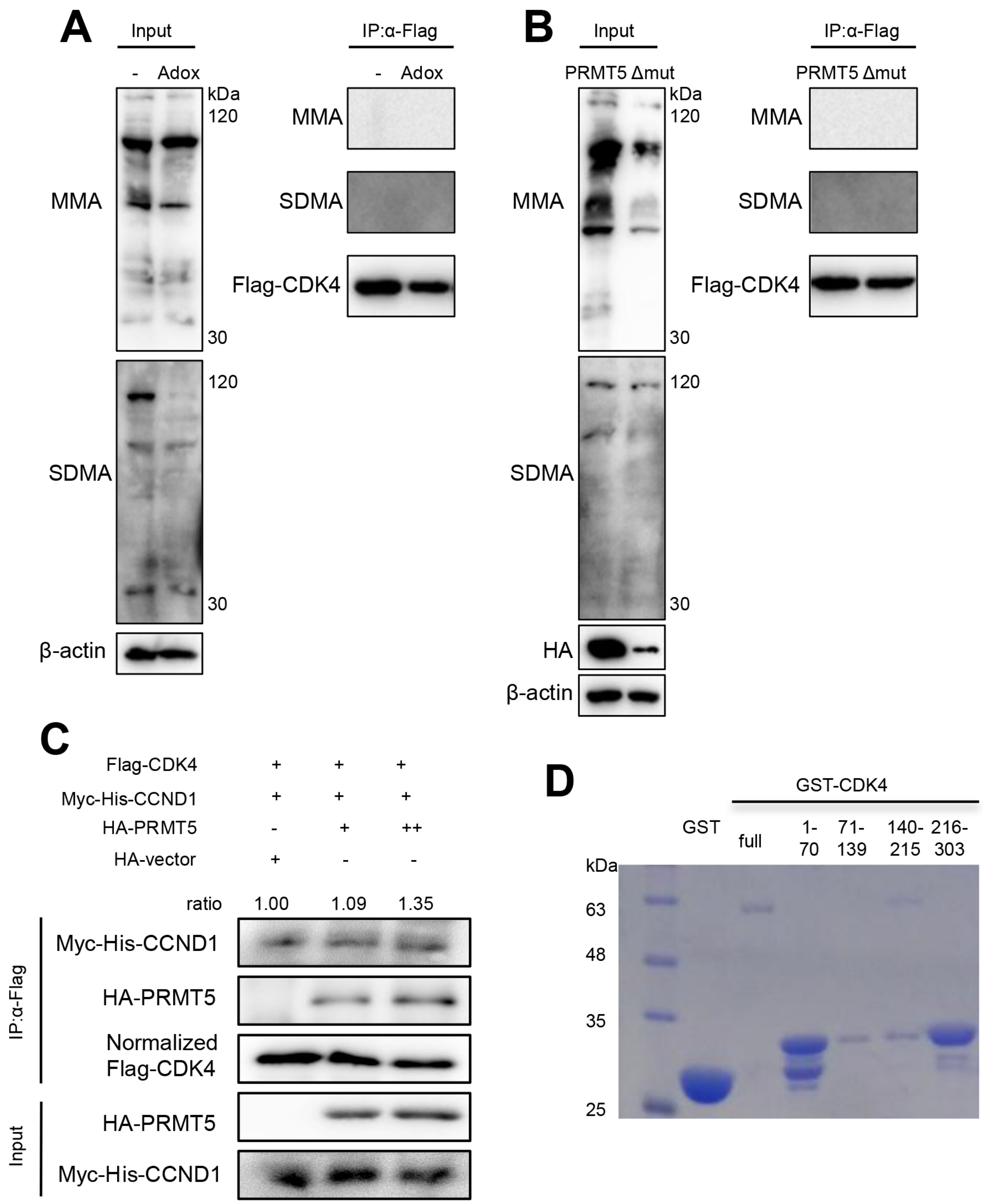

Supplementary Figure S4: The methylation of CDK4 and the competitive binding. A. HepG2 cells expressing Flag-CDK4 were treated with or without adenosine-2', 3'-dialdehyde (Adox) for 8 hours. The cell lysates were immunoprecipitated by anti-Flag M2 affinity gel, and the mono-methylated arginine (MMA) and symmetric dimethylated arginine (SDMA) levels of CDK4 were examined by Western blots. **B.** HepG2 cells were transfected with Flag-CDK4 and HA-PRMT5 enzymatic mutation Δmut (or HA-PRMT5). The Flag-CDK4 proteins were immunoprecipitated by anti-Flag M2 affinity gel, and the methylation levels of CDK4 were examined by Western blots. **C.** HEK293T cells were co-transfected with Flag-CDK4, Myc-6×His-CCND1 and HA-PRMT5 (0.4 μg or 4 μg with HA-empty vector as control). Flag-CDK4 proteins were immunoprecipitated, and Myc-His-CCND1 protein levels in the samples were examined by Western blot. **D.** GST-fused CDK4 and its fragments expressed in *E.coli* were purified and then examined by SDS-PAGE followed Coomassie blue staining.

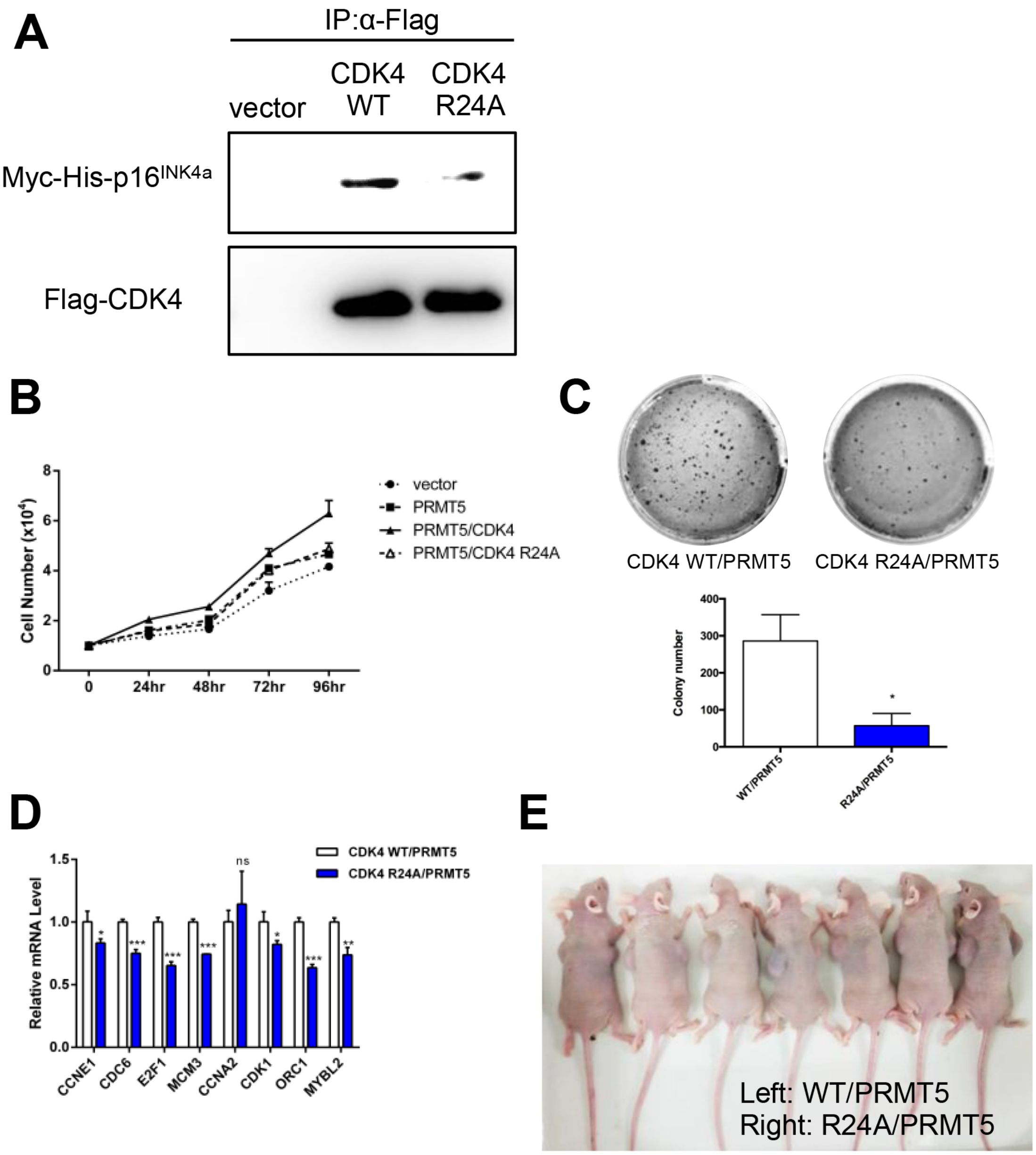

Supplementary Figure S5: CDK4 R24A inhibits HCC cell proliferation. A. HEK293T cells were co-transfected with Flag-CDK4 wild-type (R24A or Flag-empty vector) and Myc-6×His-p16^{INK4a} for 48 hours. The interaction was examined by co-IP assays followed Western blots. **B.** HuH-7 cells were transfected with HA-empty vector, HA-PRMT5, HA-PRMT5/Flag-CDK4, or HA-PRMT5/Flag-CDK4 R24A. The cell numbers were counted every 24 hours. **C.** HepG2 cells stably expressing CDK4 WT (or R24A) and PRMT5 were seeded and cultured in high or low glucose medium with agarose gel to perform colony formation assay. The pictures of crystal violet staining cells were presented, and the colony numbers were calculated and tested with t-test. $*P < 0.05$. **D.** The mRNA levels of CDK4-RB-E2F1 target genes in HepG2 cells stably expressing CDK4 WT/PRMT5 and CDK4 R24A/PRMT5 were examined by qRT-PCR. Data are presented as the mean ± SD and tested with t-test from three independent experiments. NS (no significant difference), $*P < 0.05$, $**P < 0.01$, $***P < 0.001$. **E.** Nude mice were injected with $5{\times}10^6$ HepG2 cells stably expressed CDK4 WT/PRMT5 (left side) and CDK4 R24A/PRMT5 (right side), and the mice were imaged after 22 days.
